# Supplementary material for: Above and belowground phenotypic response to exogenous auxin across Arabidopsis thaliana mutants and natural accessions varies from seedling to reproductive maturity
Source: PeerJ. 2024 Feb 9;12:e16873. doi: 10.7717/peerj.16873 (PMC10860551; doi:10.7717/peerj.16873)
Supplement: Supplemental Information 6 [file peerj-12-16873-s006.pdf]

Supplemental Materials

Supplement S1: Additional data from publicly available databases on variation in APA of genes selected for study.

Table S1 - Polyadenylation information for select auxin related genes and their respective SALK T-DNA insert mutant lines with gene information adapted from Hong et al. 2018.

Gene Type	Gene Family	Gene	Locus	PA Site Number	PA Group	SALK Line
Auxin Signal Transduction	Auxin Binding Protein	ABP1	AT4G02980	4	>2	SALK_208329C
Auxin Signal Transduction	Auxin Signaling F Box Protein	AFB3	AT1G12820	1	1+2	SALK_068787C
Auxin Signal Transduction	Auxin Response Factor	ARF1	AT1G59750	2	1+2	SALK_079046C
Auxin Signal Transduction	Auxin Response Factor	ARF11	AT2G46530	4	>2	SALK_063778C
Auxin Signal Transduction	Auxin Response Factor	ARF14	AT1G35540	0	0	SALK_049581C
Auxin Signal Transduction	Auxin Response Factor	ARF21	AT1G34410	0	0	SALK_020702C
Auxin Signal Transduction	Auxin Response Factor	ARF4	AT5G60450	3	>2	SALK_070506C
Auxin Signal Transduction	Indole-3-Acetic Acid Inducible	IAA15	AT1G80390	0	0	SALK_150265C
Auxin Signal Transduction	Indole-3-Acetic Acid Inducible	IAA33	AT5G57420	0	0	SALK_134724C
Auxin Signal Transduction	Indole-3-Acetic Acid Inducible	IAA5	AT1G15580	2	1+2	SALK_112553C
Auxin Signal Transduction	Indole-3-Acetic Acid Inducible	IAA8	AT2G22670	1	1+2	SALK_202296C
Auxin Transport	Like Auxin Resistant	LAX2	AT2G21050	1	1+2	SALK_208379C
Auxin Transport	PIN-Formed	PIN7	AT1G23080	3	>2	SALK_048791C
Auxin Transport	PIN-Formed	PIN8	AT5G15100	0	0	SALK_204263C
Auxin Synthesis Metabolism	SuperRoot	SUR1	AT2G20610	1	1+2	SALK_113460C

We gathered genome annotation data from Araport11 (Cheng et al. 2017), polyadenylation cluster location data from the PlantAPAdb (Zhu et al. 2020), and T-DNA insert location data from T-DNA Express (signal.salk.edu) to visualize the variation across loci of interest.

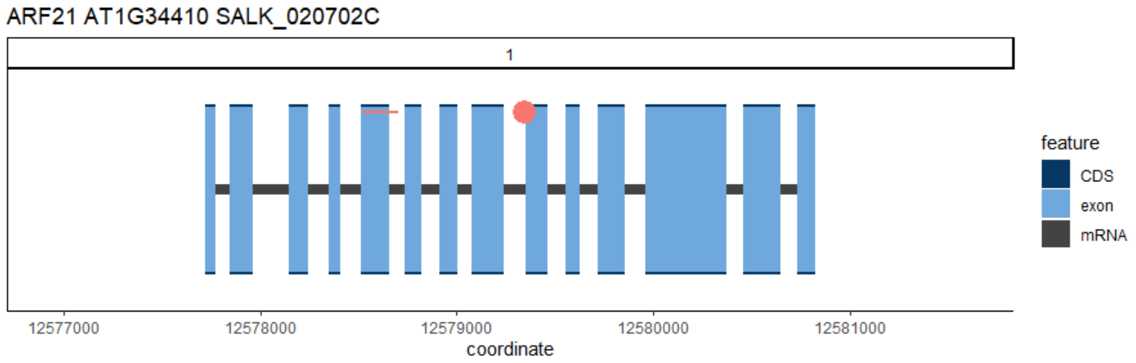

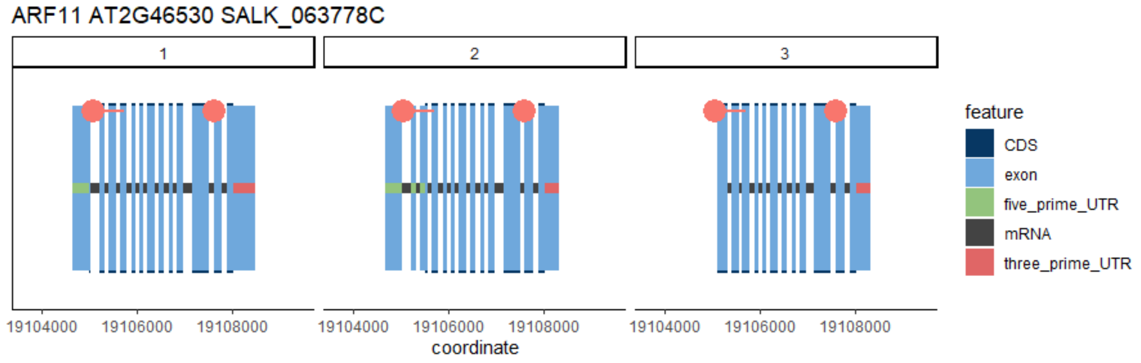

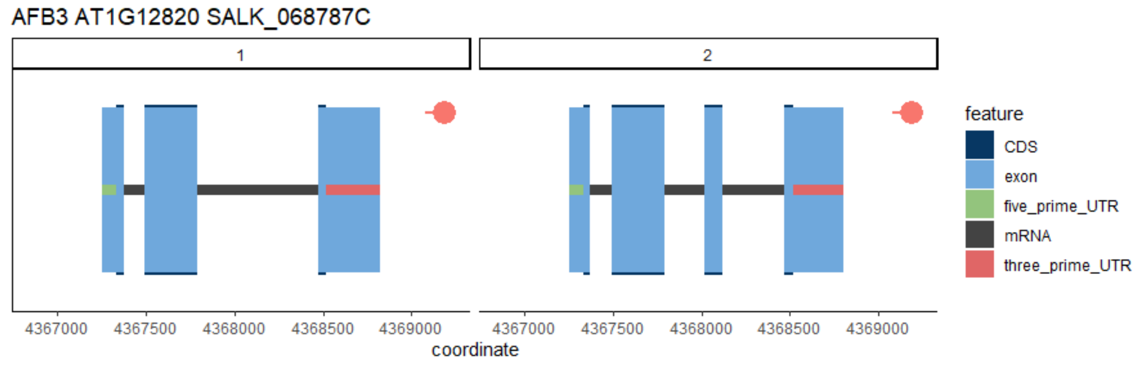

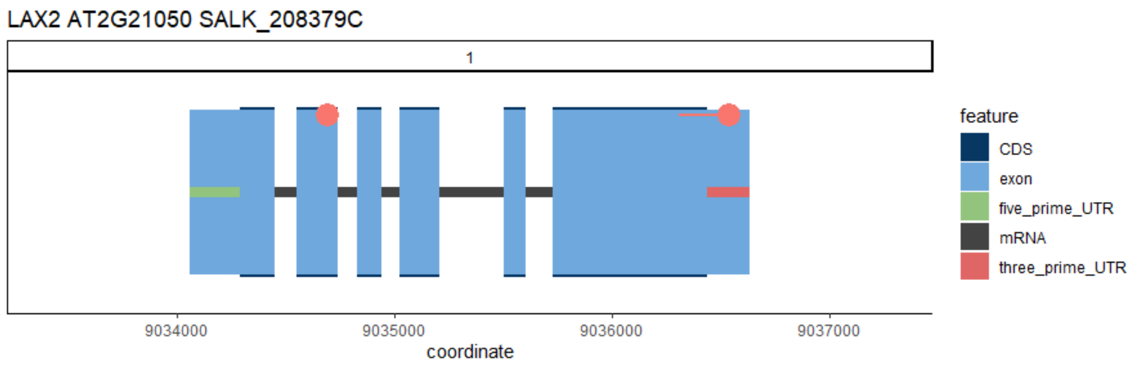
LAX2 AT2G21050 SALK_208379C
1
feature
CDS
exon
five_prime_UTR
mRNA
three_prime_UTR
9034000
9035000
9036000
9037000
coordinate

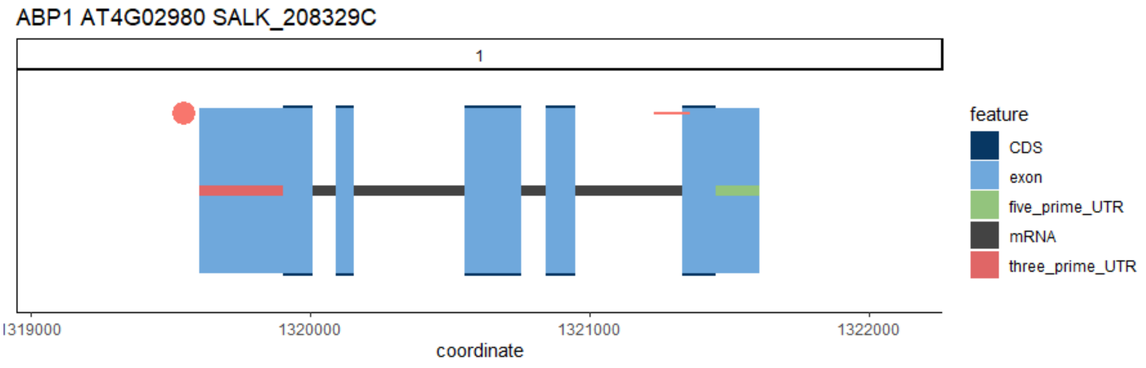
ABP1 AT4G02980 SALK_208329C
1
feature
CDS
exon
five_prime_UTR
mRNA
three_prime_UTR
1320000
1321000
1322000
coordinate

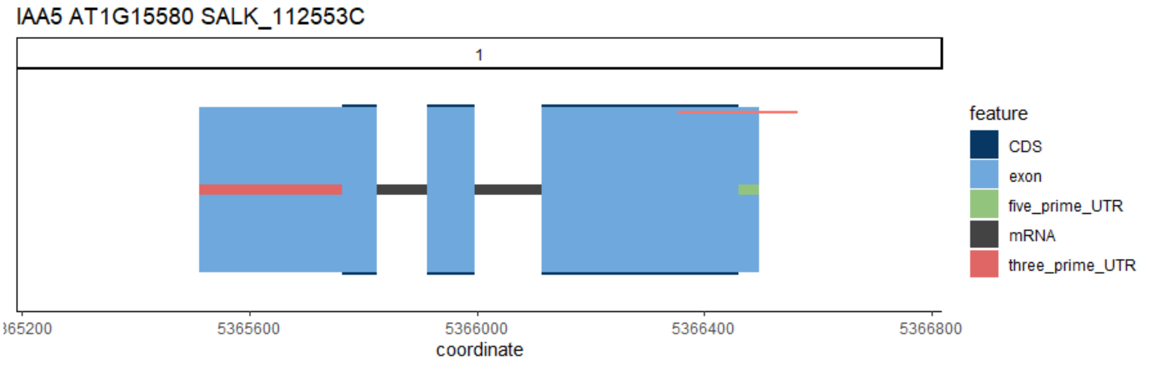
IAA5 AT1G15580 SALK_112553C
1
feature
CDS
exon
five_prime_UTR
mRNA
three_prime_UTR
5365600
5366000
5366400
5366800
coordinate

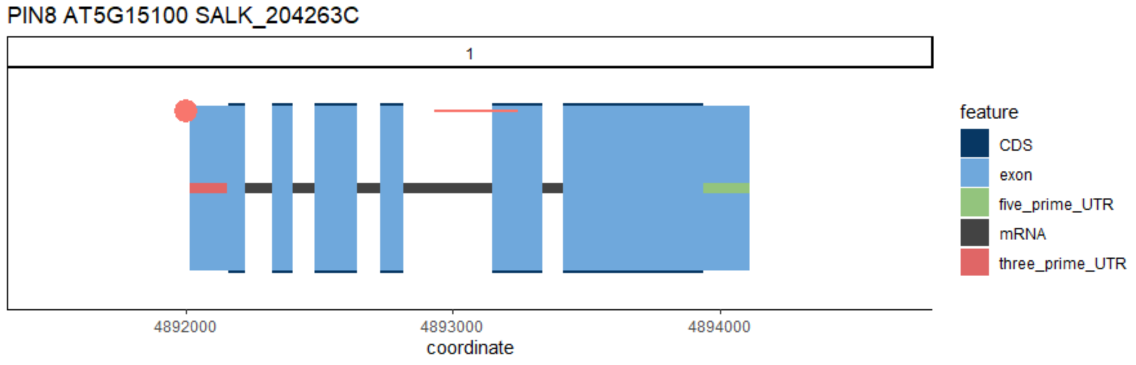
PIN8 AT5G15100 SALK_204263C
1
feature
CDS
exon
five_prime_UTR
mRNA
three_prime_UTR
4892000
4893000
4894000
coordinate

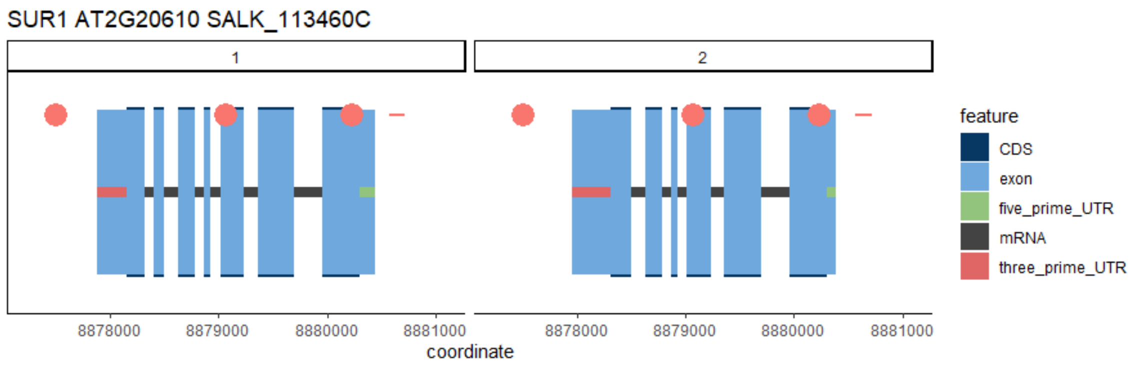
SUR1 AT2G20610 SALK_113460C
1
2
8878000
8879000
8880000
8881000
coordinate
feature
CDS
exon
five_prime_UTR
mRNA
three_prime_UTR

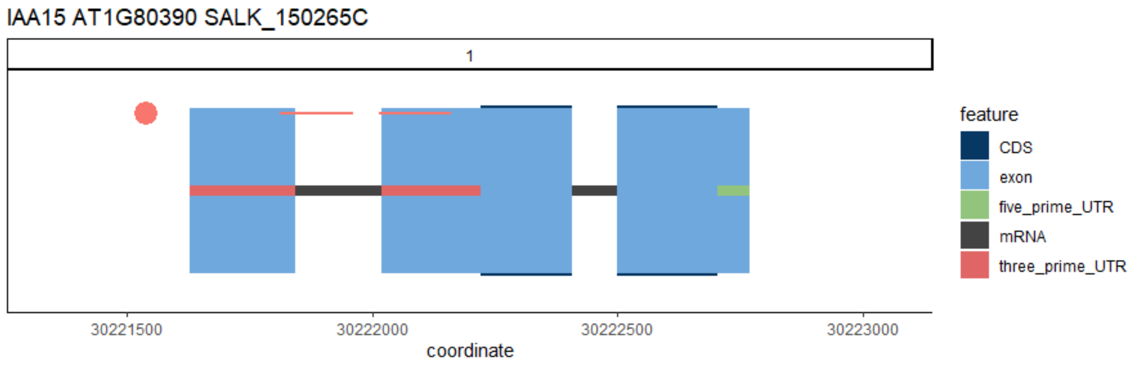
IAA15 AT1G80390 SALK_150265C
1
30221500
30222000
30222500
30223000
coordinate
feature
CDS
exon
five_prime_UTR
mRNA
three_prime_UTR

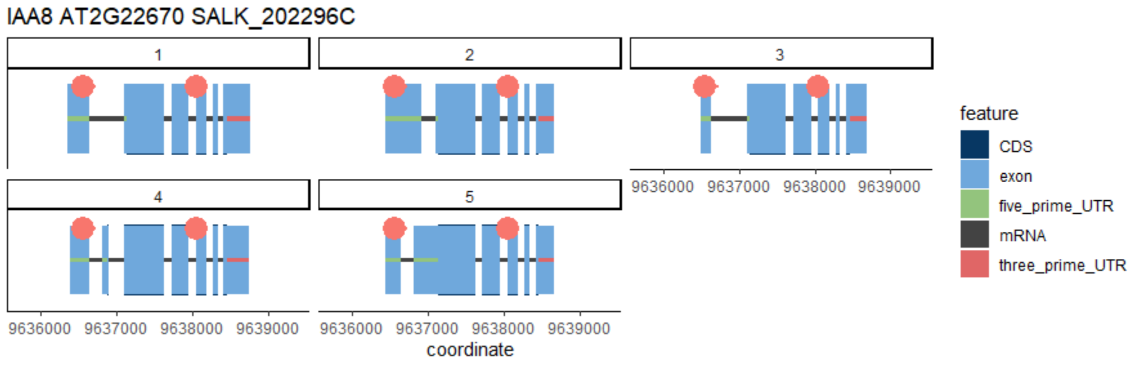
IAA8 AT2G22670 SALK_202296C
1
2
3
4
5
9636000
9637000
9638000
9639000
coordinate
feature
CDS
exon
five_prime_UTR
mRNA
three_prime_UTR

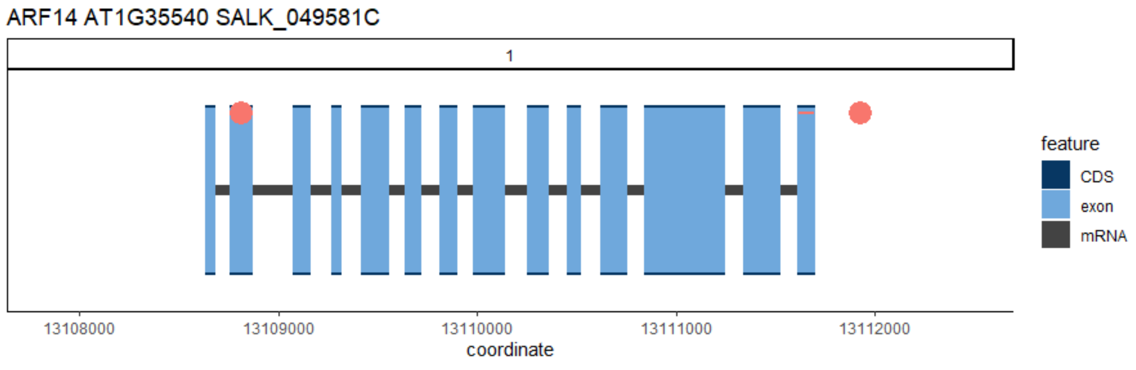
ARF14 AT1G35540 SALK_049581C
1
13108000
13109000
13110000
13111000
13112000
coordinate
feature
CDS
exon
mRNA

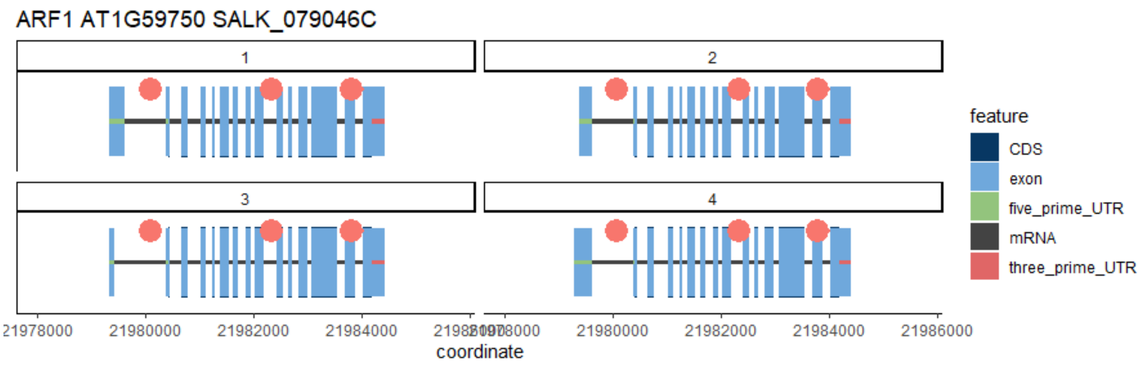
ARF1 AT1G59750 SALK_079046C
1
2
3
4
feature
CDS
exon
five_prime_UTR
mRNA
three_prime_UTR
21978000
21980000
21982000
21984000
21980000
21982000
21984000
21986000
coordinate

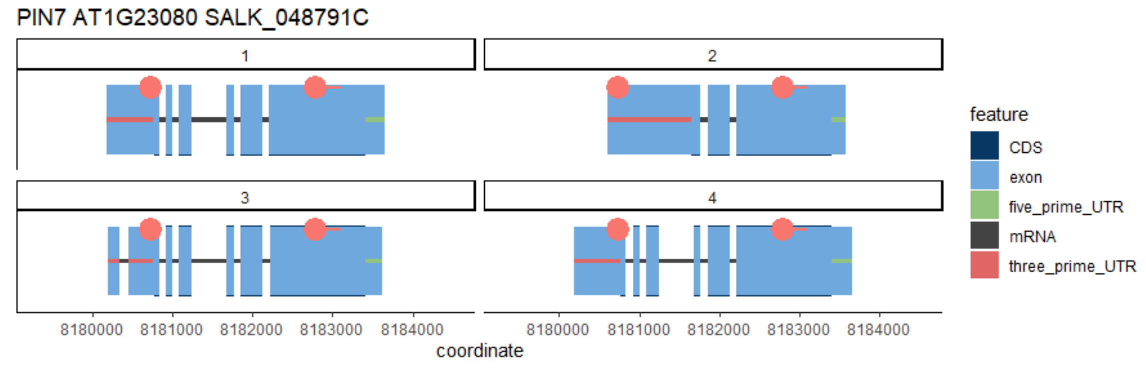
PIN7 AT1G23080 SALK_048791C
1
2
3
4
feature
CDS
exon
five_prime_UTR
mRNA
three_prime_UTR
8180000
8181000
8182000
8183000
8184000
8180000
8181000
8182000
8183000
8184000
coordinate

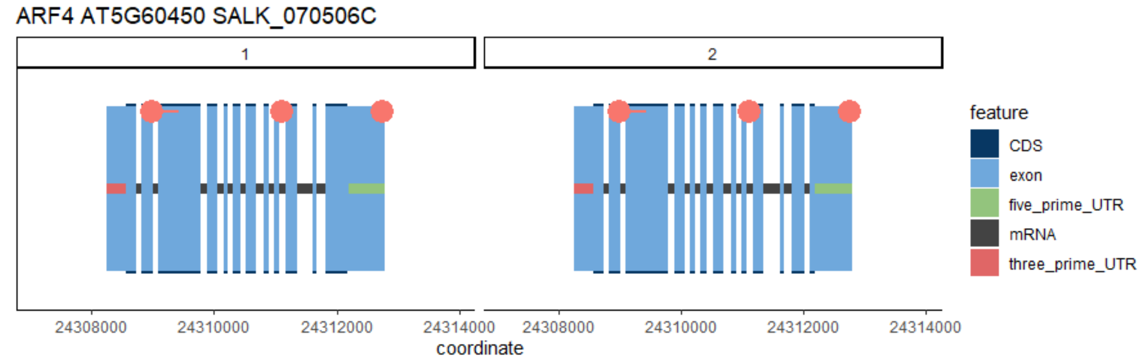
ARF4 AT5G60450 SALK_070506C
1
2
feature
CDS
exon
five_prime_UTR
mRNA
three_prime_UTR
24308000
24310000
24312000
24314000
24308000
24310000
24312000
24314000
coordinate

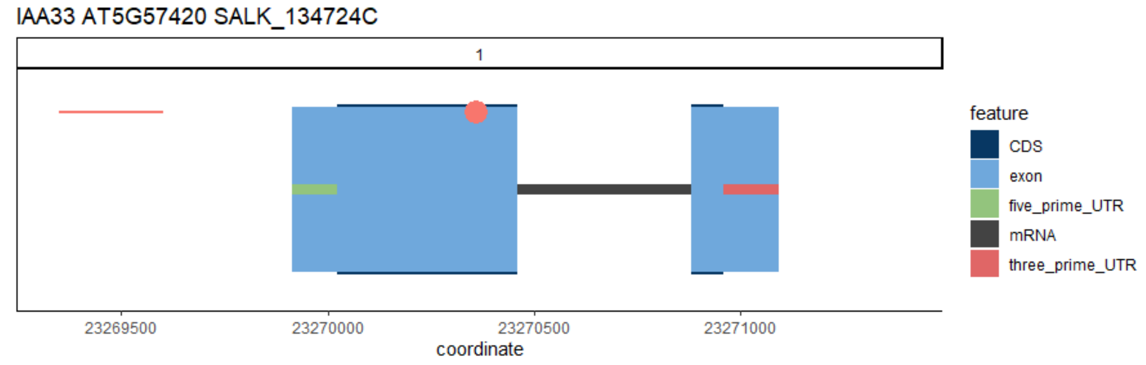
IAA33 AT5G57420 SALK_134724C
1
feature
CDS
exon
five_prime_UTR
mRNA
three_prime_UTR
23269500
23270000
23270500
23271000
coordinate

Figure S1 - Annotated transcripts of focal genes in the study. Titles of plots include the gene name, its locus, and corresponding SALK insert mutant line available in TAIR11. Different plots underneath the same gene, locus, and SALK line represent different transcripts described in the Araport11 genome annotation. Pink circles represent polyadenylation clusters recognized by studies within the PlantAPAdb. Pink lines represent the location of SALK T-DNA inserts. Multiple inserts within one annotation represent multiple inserts detected at the SALK institute. The lines used for this investigation, however, have been confirmed by unPAK (See Rutter et al. 2019) to be homozygous single inserts.

Supplement S2: Synthesis of Existing Auxin Root Assay Literature

We searched the Web of Science database in September 2020 using the search term "lateral root development gene auxin" to obtain articles studying the role of auxin in lateral root development in the window from 1980 – Sept 2020. We then completed a set of filters (Figure S1) based on a series of criteria. Papers were retained if they mentioned lateral roots within the abstract and if they noted the specific loci of genes studied. Our goal for quantitative synthesis of publications was to determine the developmental stage at which the genes frequently studied in the literature, the substrate in which plants were grown, the age at which roots were phenotype, and the method and traits used for root phenotyping.

The results of this synthesis informed our methods by identifying gaps in the literature in the developmental stage of root assays. Lateral root traits of auxin-related mutants were measured after a mean of 11 days (SD=5.0; Fig. S3), with 94% of studies growing plants on an agar substrate (Fig. S4). These findings guided our choices in determining when plants were examined (14d, 21d, and maturity) and the substrates on which they were grown (sand and potting mix).

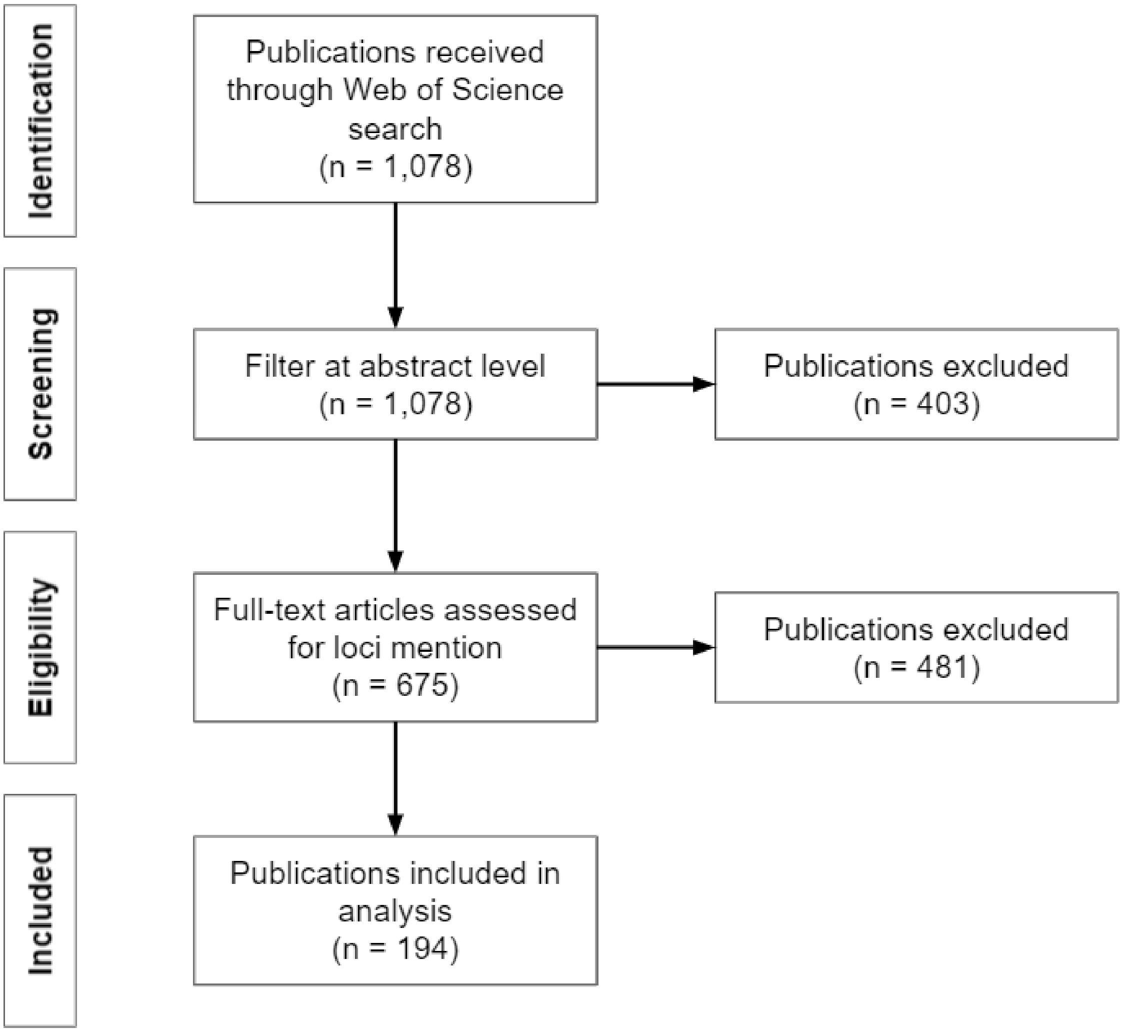

Figure S2 - Flow chart diagram of literature identification, screening and selection adapted from (Duenas et al. 2021) based on PRISMA (Preferred Reporting Items for Systematic Reviews and Meta-Analyses) guidelines (Moher et al. 2009).

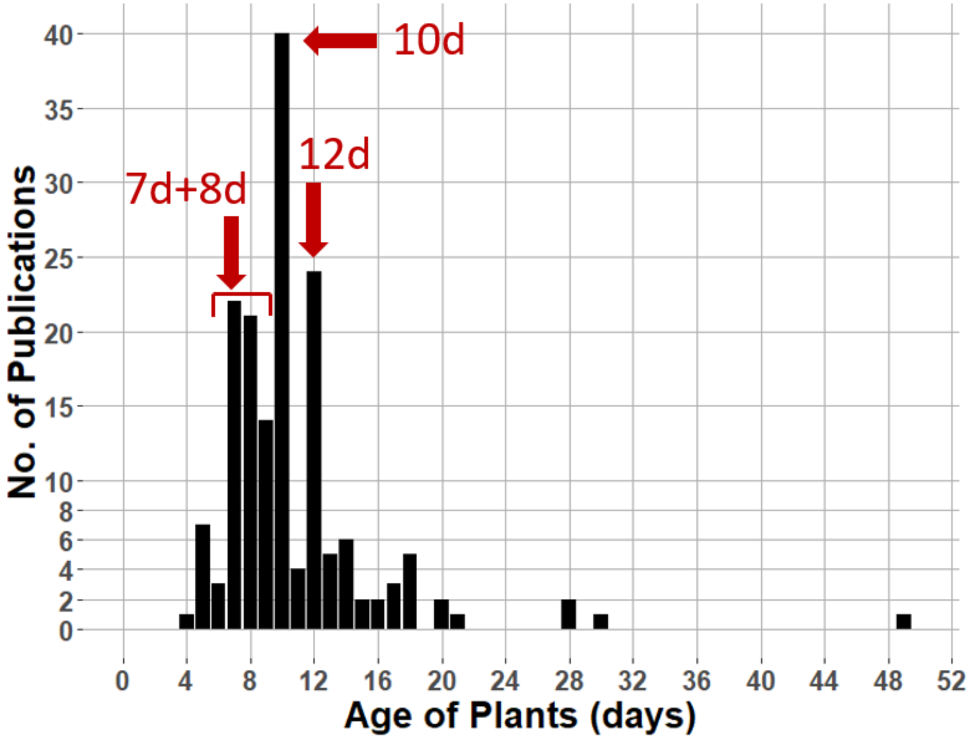

Figure S3 - Time of lateral root measurements noted in the publications from the quantitative synthesis of the literature (n=166, mean days old plant = 10.8, +/- 1 SD=5.03).

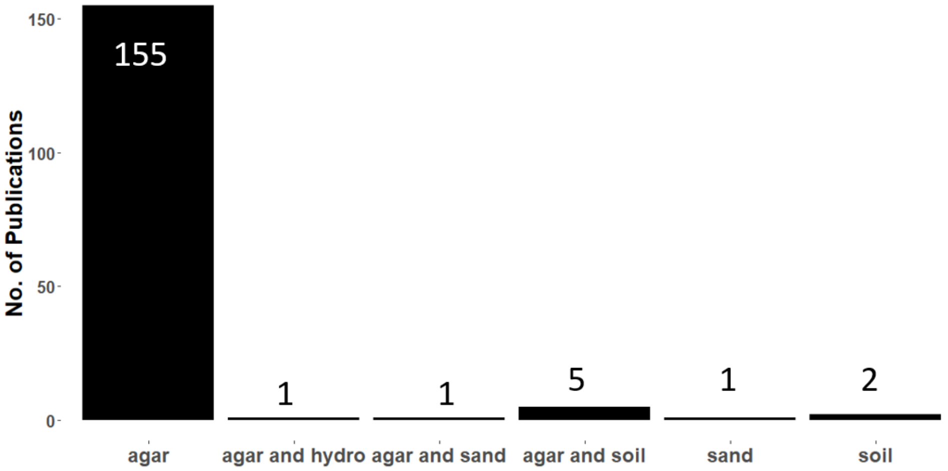

Figure S4 - Frequency substrates (media) used across publications (n=166) in the

Table S2 - Analysis of variance examining the fixed effects of hormone treatment, genotype, and their interaction in 14d SALK insert mutant seedlings. Values are F-statistic results of linear models, and significance levels are indicated by p<0.001=***, p<0.01=**, p<0.05=*, p>0.05=ns with exact p-values noted in parentheses. Belowground mass and LR density have been square root transformed and aboveground mass log-transformed to meet ANOVA assumptions.

Fixed Effect	Rosette Diam.	Root Length	Below Mass	Above Mass	LR Density
Treatment<br>df=1	22.3***<br>(5.5e-06)	11.2**<br>(0.0011)	4.3*<br>(0.041)	32.8***<br>(6.1e-08)	97.4***<br>(<2e-16)
Genotype<br>df=14	4.5***<br>(1.2e-06)	2.6**<br>(0.0023)	3.7***<br>(3.5e-05)	3.5***<br>(6.5e-05)	1 ns<br>(0.43)
Treatment*Genotype<br>df=14	0.7 ns<br>(0.74)	0.6 ns<br>(0.90)	0.6 ns<br>(0.83)	0.6 ns<br>(0.83)	0.8 ns<br>(0.69)

Table S3 - Mean and one standard error of seedling traits in control and auxin hormone treatments at 14 and 21 days.

Collection Time	Treatment	Rosette Diam. (mm) ±SE	Root Length (cm) ±SE	Below Mass (mg) ±SE	Above Mass (mg) ±SE	LR Density (#/cm) ±SE
14d	Control	14.5±0.5	38.2±3.3	2.2±0.1	0.28±0.01	3.3±0.2
14d	Auxin	11.7±0.4	29.1±2.1	1.3±0.1	0.25±0.01	4.5±0.3
21d	Control	24.5±0.5	128.6±6.2	11.8±0.5	1.13±0.54	4.9±0.2
21d	Auxin	23.3±0.4	127.1±6.0	7.0±0.3	1.16±0.31	6.5±0.2

Table S4 - Analysis of variance examining the fixed effects of hormone treatment, genotype, and their interaction in 21d SALK insert mutant seedlings. Values are F-statistic results of linear models, and significance levels are indicated by p<0.001=***, p<0.01=**, p<0.05=*, p>0.05=ns with exact p-values noted in parentheses. Belowground mass, aboveground mass, and LR density have been square root transformed and rosette diameter log-transformed to meet assumptions.

Fixed Effect	Rosette Diam.	Root Length	Below Mass	Above Mass	LR Density
Treatment<br>df=1	1.9 ns<br>(0.1730)	0.03 ns<br>(0.8684)	0.2 ns<br>(0.64722)	45.5***<br>(4.48e-10)	27.9***<br>(5.715e-07)
Genotype<br>df=14	0.9 ns<br>(0.5697)	1.0 ns<br>(0.4765)	1.6 ns<br>(0.07974)	1.3 ns<br>(0.1983)	1.3 ns<br>(0.1892)
Treatment*Genotype<br>df=14	0.6 ns<br>(0.8372)	0.6 ns<br>(0.8834)	0.9 ns<br>(0.60353)	0.9 ns<br>(0.6055)	0.5 ns<br>(0.9140)

Table S5 - Analysis of variance examining the fixed effects of hormone treatment, genotype, and their interaction in mature SALK insert mutants. Values are F-statistic results of linear models, and significance levels are indicated by p<0.001=***, p<0.01=**, p<0.05=*, p>0.05=ns with exact p-values noted in parentheses. Inflorescence height has been log-transformed and fruit number, days to bolt, and days to flower square root transformed to meet assumptions. The tray random effect term refers to plant plug trays and was included to account for random differences across our block design. Likelihood ratio test statistics are reported for random effects with exact p-values in parentheses.

Fixed Effect	Diam. at Bolt	Root Length	Below Mass	Above Mass	Infl. Height	Infl. Branchin
Treatment df=1	15.2*** (0.00015)	45.6*** (3.9e-10)	84.5*** (5.6e-16)	15.6*** (0.00013)	10.3** (0.0017)	0.8 ns (0.38)
Genotype df=14	0.8 ns (0.67)	1.5 ns (0.11)	1.3 ns (0.19)	1.1 ns (0.37)	1.9* (0.035)	1.0 ns (0.42)
Treat.*Geno. df=14	1.5 ns (0.11)	1.4 ns (0.16)	1.6 ns (0.084)	1.5 ns (0.11)	1.7 ns (0.073)	1.2 ns (0.27)
**Rand. Effect**	13.7	26.7	32.3	36.0	18.4	18.0
Tray df=1	*** (0.00021)	*** (2.4e-07)	*** (1.3e-08)	*** (2.0e-09)	*** (1.8e-05)	*** (2.2e-05)

Fixed Effect	Fruit Number	Avg. Fruit Length	Days to Bolt	Days to Flower	Days to Mature	
Treatment df=1	0.2 ns (0.69)	27*** (7.3e-07)	24.6*** (2.0e-06)	16.3*** (9.2e-05)	11.1*** (0.0011)	
Genotype df=14	1.3 ns (0.21)	1.9* (0.026)	1.8* (0.037)	2.2** (0.0091)	2.1* (0.015)	
Treat.*Geno. df=14	1.3 ns (0.23)	1.4 ns (0.17)	1.3 ns (0.20)	2.1* (0.017)	1.3 ns (0.24)	
**Rand. Effect**	46.0	1.8	2.2	3.9	4.9	
Tray df=1	*** (1.2e-11)	ns (0.18)	ns (0.14)	* (0.049)	* (0.027)	

Table S6 - Mean and one standard error of traits measured in mature adult plants in control and auxin hormone treatments. Bold mean values indicate a significant difference between treatments.

Collection Time	Treatment	Diam. at Bolt (mm) ±SE	Root Length (cm) ±SE	Below Mass (mg) ±SE	Above Mass (mg) ±SE	Germination Day ±SE	Infl. Height (cm) ±SE	Infl. Branch ±SE
Mature	Control	**20.5±0.5**	**233.6±15.0**	**2.4±0.1**	**33.2±1.6**	2.6±0.1	**21.5±0.5**	3.4±0.3
Mature	Auxin	**23.5±0.6**	**366.4±15.3**	**4.3±0.2**	**41.4±1.6**	2.7±0.1	**23.8±0.4**	3.1±0.2
**Collection Time**	**Treatment**	**Fruit Number ±SE**	**Avg. Fruit Length (mm) ±SE**	**Fitness (fruit no. / length) ±SE**	**Days to Bolt ±SE**	**Days to Flower ±SE**	**Days to Mature ±SE**	
Mature	Control	22.7±1.0	**10.5±0.2**	248.8±12.1	**25.0±0.6**	**29.1±0.6**	**44.5±0.5**	
Mature	Auxin	21.7±0.9	**11.7±0.1**	257.0±11.9	**28.0±0.3**	**31.5±0.3**	**47.0±0.3**	

Table S7 - Analysis of variance examining the fixed effects of genotype, and developmental stage by genotype interaction for 14d and 21d seedlings. Values are F-statistic results of linear models, and significance levels are indicated by p<0.001=***, p<0.01=**, p<0.05=*, p>0.05=ns with exact p-values noted in parentheses. Belowground mass, aboveground mass, and LR density have been square root transformed and rosette diameter as well as root length have been log-transformed to meet assumptions. Likelihood ratio test statistics are reported for random effects with exact p-values in parentheses.

Control:					
**Fixed Effect**	**Rosette Diam.**	**Root Length**	**Below Mass**	**Above Mass**	**LR Density**
Genotype<br>df=20	3.1***<br>(2.9e-05)	3.4***<br>(4.4e-06)	3.2***<br>(1.6e-05)	3.3***<br>(7.6e-06)	0.9 ns<br>(0.65)
Developmental Stage*Genotype<br>df=20	1.1 ns<br>(0.36)	1.0 ns<br>(0.47)	1.5 ns<br>(0.10)	0.9 ns<br>(0.53)	0.7 ns<br>(0.86)
Auxin:					
**Fixed Effect**	**Rosette Diam.**	**Root Length**	**Below Mass**	**Above Mass**	**LR Density**
Genotype<br>df=20	2.9***<br>(6.8e-05)	2.7***<br>(0.00026)	2.8***<br>(0.00011)	1.5 ns<br>(0.079)	1.2 ns<br>(0.24)
Developmental Stage*Genotype<br>df=20	1.3 ns<br>(0.19)	1.0 ns<br>(0.52)	1.1 ns<br>(0.31)	0.8 ns<br>(0.66)	2.1**<br>(0.0059)

Table S8 – Analysis of Variance examining the fixed effects of hormone treatment, genotype, and their interaction in mature plants. All traits have been scaled according to their developmental stage and treatment such that developmental stage*genotype effects represent changes in rank order of genotypes but not different magnitudes or direction of difference. Values are F-statistic results of linear models, and significance levels are indicated by p<0.001=***, p<0.01=**, p<0.05=*, p>0.05=ns with exact p-values noted in parentheses. The tray random effect term refers to plant plug trays and was included to account for random differences across our block design. Likelihood ratio test statistics are reported with exact p-values in parentheses.

Control:<br>**Fixed Effect**	**Root Length**	**Below Mass**	**Above Mass**
Developmental Stage (df=2)	0.1 ns (0.94)	0.03 ns (0.97)	0.1 ns (0.92)
Genotype (df=20)	3.8*** (1.9e-07)	2.6*** (0.00034)	2.5*** (0.00054)
Develop. Stage *Genotype (df=40)	1.1 ns (0.3)	1.8** (0.0045)	1.6* (0.021)
**Rand. Effect**<br>Tray (df=1)	27.7*** (1.4e-07)	5.2* (0.022)	7.1** (0.0078)
Auxin:<br>**Fixed Effect**	**Root Length**	**Below Mass**	**Above Mass**
Developmental Stage (df=2)	0.089 ns (0.92)	0.1 ns (0.90)	0.14 ns (0.87)
Genotype (df=20)	4.6*** (1.2e-09)	2.6*** (0.00024)	1.3 ns (0.19)
Developmental Stage *Genotype (df=40)	1.4 ns (0.078)	1.6* (0.012)	1.1 ns (0.29)
**Rand. Effect**<br>Tray (df=1)	124.2*** (<2.2e-16)	71.2 *** (<2.2e-16)	80.3 *** (<2.2e-16)

Table S9 – Analysis of Variance examining the fixed effects of developmental stage, APA group (0, 1+2, or >2) and their interaction in mature SALK insert mutants. All traits have been scaled according to their developmental stage and treatment such that developmental stage*APA group effects represent changes in rank order of APA groups but not different magnitudes or direction of difference. Values are F-statistic results of linear models, and significance levels are indicated by p<0.001=***, p<0.01=**, p<0.05=*, p>0.05=ns with exact p-values note in parentheses. The tray random effect term refers to plant plug trays and was included to account for the block design. Likelihood ratio test statistics are reported with exact p-values in parentheses.

Control:<br>**Fixed Effect**	**Root Length**	**Below Mass**	**Above Mass**
Developmental Stage (df=2)	0.06 ns (0.94)	0.03 ns (0.97)	0.03 ns (0.97)
APA Group (df=2)	0.04 ns (0.96)	0.06 ns (0.94)	0.23 ns (0.79)
Develop. Stage *APA Group (df=4)	1.8 ns (0.13)	2.2 ns (0.073)	1.7 ns (0.15)
**Rand. Effect**<br>Tray (df=1)	15.5*** (8.2e-05)	0.55 ns (0.46)	4.6* (0.031)
Auxin:<br>**Fixed Effect**	**Root Length**	**Below Mass**	**Above Mass**
Developmental Stage (df=2)	0.1 ns (0.91)	0.04 ns (0.95)	0.04 ns (0.97)
APA Group (df=2)	3.3* (0.040)	7.1** (0.0010)	4.9** (0.0082)
Developmental Stage *APA Group (df=4)	0.7 ns (0.60)	0.8 ns (0.52)	1.6 ns (0.18)
**Rand. Effect**<br>Tray (df=1)	94.8*** (<2.2e-16)	50.2*** (1.4e-12)	72.0*** (<2.2e-16)

Table S10 - Mean and one standard error of root length, belowground mass, and aboveground mass of all PA groups (0 = no APA sites, 1+2 = 1 or 2 APA sites, >2 = more than 2 APA sites in mutant gene) in control and auxin treatments across all developmental stages.

Collection Time	Treatment	PA Group	Root Length (cm) ±SE	Below Mass (mg) ±SE	Above Mass (mg) ±SE
14d	control	0	40.4±3.4	0.31±0.02	2.28±0.18
14d	control	1+2	39.8±3.2	0.29±0.02	2.36±0.19
14d	control	>2	33.7±2.9	0.25±0.02	1.87±0.20
14d	IAA	0	29.2±2.7	0.24±0.02	1.30±0.10
14d	IAA	1+2	33.2±4.1	0.29±0.02	1.42±0.13
14d	IAA	>2	22.4±3.1	0.19±0.02	1.02±0.11
21d	control	0	119.1±9.2	0.99±0.07	10.88±0.79
21d	control	1+2	127.8±10.3	1.18±0.09	12.09±0.94
21d	control	>2	142.0±13.2	1.26±0.11	12.52±1.11
21d	IAA	0	114.5±7.6	1.04±0.06	6.33±0.33
21d	IAA	1+2	137.2±11.1	1.35±0.10	8.02±0.59
21d	IAA	>2	129.2±12.2	1.08±0.10	6.38±0.63
fruit	control	0	254.0±26.7	2.59±0.26	36.20±2.99
fruit	control	1+2	211.8±22.3	2.22±0.19	30.81±2.45
fruit	control	>2	239.3±30.3	2.59±0.33	32.83±3.28
fruit	IAA	0	335.5±25.4	4.02±0.26	38.69±2.63
fruit	IAA	1+2	385.7±23.0	4.44±0.27	42.29±2.48
fruit	IAA	>2	375.7±33.3	4.29±0.34	43.54±3.33

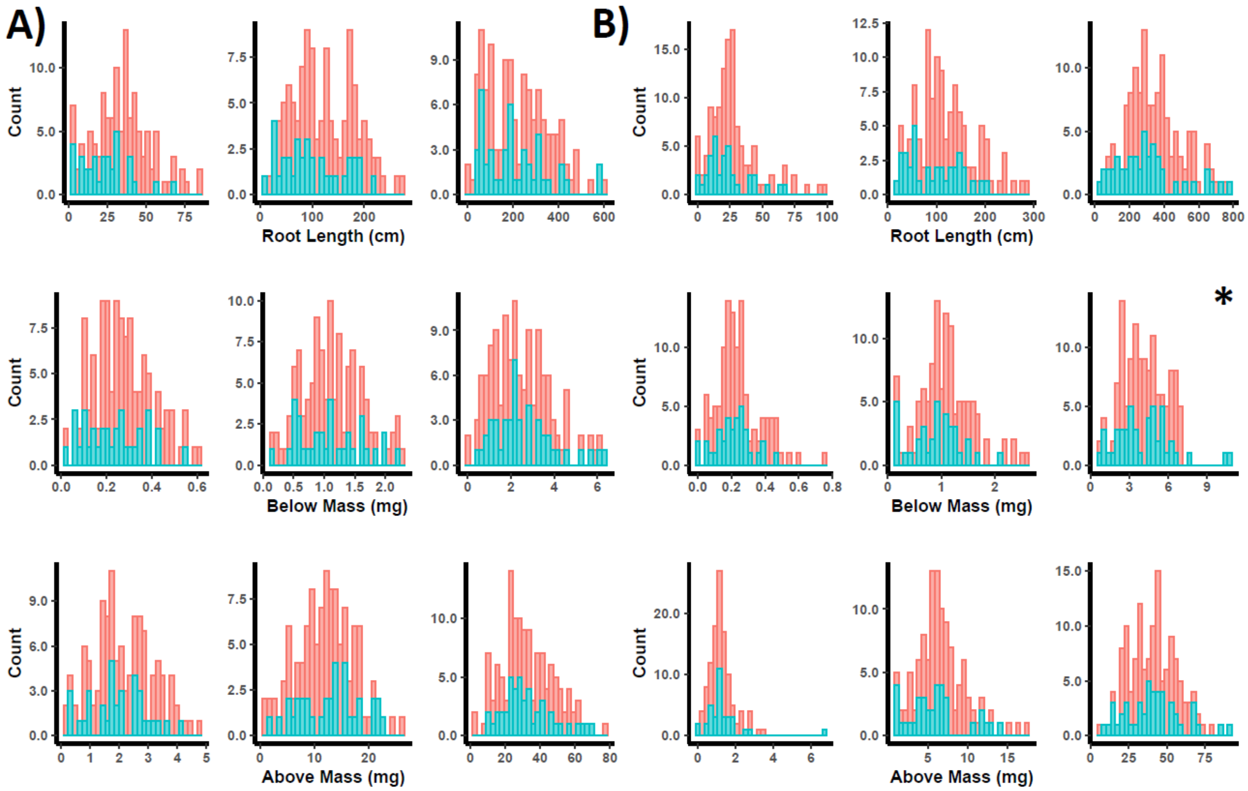

Figure S5 - Distribution of major growth traits in both treatments (control in A and auxin in B) across all life stages (14d, 21d, and mature adult left to right) for insert mutants (pink) and natural accessions (blue). Significant difference in variation between natural accessions and insert mutants determined via Levene's tests are noted by an * (df = 1, F value = 3.98, p=0.0481).

References

Cheng, C. Y., Krishnakumar, V., Chan, A. P., Thibaud-Nissen, F., Schobel, S., & Town, C. D. (2017). Araport11: a complete reannotation of the *Arabidopsis thaliana* reference genome. *The Plant Journal*, *89*(4), 789-804.

Duenas, M. A., Hemming, D. J., Roberts, A., & Diaz-Soltero, H. (2021). The threat of invasive species to IUCN-listed critically endangered species: A systematic review. *Global Ecology and Conservation*, *26*, e01476.

Hong, L., Ye, C., Lin, J., Fu, H., Wu, X., & Li, Q. Q. (2018). Alternative polyadenylation is involved in auxin-based plant growth and development. *The Plant Journal*, *93*(2), 246-258.

Moher, D., Liberati, A., Tetzlaff, J., Altman, D. G., & PRISMA Group*. (2009). Preferred reporting items for systematic reviews and meta-analyses: the PRISMA statement. *Annals of Internal Medicine*, *151*(4), 264-269.

Rutter, M. T., Murren, C. J., Callahan, H. S., Bisner, A. M., Leebens-Mack, J., Wolyniak, M. J., & Strand, A. E. (2019). Distributed phenomics with the unPAK project reveals the effects of mutations. *The Plant Journal*, *100*(1), 199-211.

Zhu, S., Ye, W., Ye, L., Fu, H., Ye, C., Xiao, X., ... & Wu, X. (2020). PlantAPAdb: a comprehensive database for alternative polyadenylation sites in plants. *Plant Physiology*, *182*(1), 228-242.
